# The Portuguese version of “The Utrecht questionnaire for outcome assessment in aesthetic rhinoplasty”: validation and clinical application^☆^

**DOI:** 10.1016/j.bjorl.2017.11.007

**Published:** 2017-12-26

**Authors:** Francisco Rosa, Peter J.F.M. Lohuis, João Almeida, Mariline Santos, Jorge Oliveira, Cecília Almeida e Sousa, Miguel Ferreira

**Affiliations:** a Centro Hospitalar do Porto, Departamento de Otorrinolaringologia e Cirurgia de Cabeça e Pescoço, Porto, Portugal; b Diakonessen Hospital, Center for Facial Plastic and Reconstructive Surgery, Department of Otolaryngology/Head and Neck Surgery, Utrecht, The Netherlands

**Keywords:** Surveys and questionnaires, Rhinoplasty, Quality of life, Plastic surgery, Patient satisfaction, Pesquisas e questionários, Rinoplastia, Qualidade de vida, Cirurgia plástica, Satisfação do paciente

## Abstract

**Introduction:**

The evaluation of surgical outcomes measured by patient satisfaction or quality of life is very important, especially in plastic surgery. There is increasing interest in self-reporting outcomes evaluation in plastic surgery.

**Objective:**

The aim of this study was to perform the translation, cross-cultural adaptation and validation of “The Utrecht questionnaire for outcome assessment in aesthetic rhinoplasty” from English to Portuguese.

**Methods:**

Retrospective study involving 50 patients undergoing to rhinoplasty comparing the preoperative period with the current postoperative situation (minimum 6 months and maximum 24 months postoperatively). Statistical analysis was performed to assess internal consistency, test–retest reliability, validity and responsiveness.

**Results:**

No patients received a negative score on the visual analogue scale comparing preoperative and postoperative appearance. The postoperative improvement on the visual analogue scale revealed a Gaussian curve of normal distribution with a mean improvement of 4.44 points. The test–retest reliability showed a positive correlation between the postoperative response and the same questionnaire repeated ninety-six hours later. The internal consistency was high (Cronbach's alpha value: Preoperative = 0.88; Postoperative = 0.86). The authors observed a significant improvement in response for all individual questions in the postoperative phase as compared with preoperative situation (*t*-student test – *p* < 0.05).

**Conclusion:**

The Portuguese version of “The Utrecht questionnaire for outcome assessment in aesthetic rhinoplasty” is a valid instrument to assess patients’ outcomes following rhinoplasty surgery.

## Resumo

**Introdução:**

A avaliação do resultado cirúrgico medido pela satisfação do paciente ou qualidade de vida é muito importante, especialmente na cirurgia plástica, uma especialidade na qual há um crescente interesse na avaliação de resultados por auto-relato.

**Objetivo:**

O objetivo deste estudo foi realizar a tradução, a adaptação transcultural e a validação do questionário “*The Utrecht questionnaire for outcome assessment in aesthetic rhinoplasty*”, do inglês para português.

**Método:**

Estudo retrospectivo envolvendo 50 pacientes submetidos a rinoplastia comparando o período pré-operatório com a situação atual (mínimo de 6 meses e máximo de 24 meses de pós-operatório). A análise estatística foi realizada para avaliar a consistência interna, confiabilidade teste-reteste, validade e capacidade de resposta.

**Resultados:**

Nenhum paciente recebeu pontuação negativa na escala visual analógica ao comparar a aparência pré- e pós-operatória. A melhora pós-operatória na escala visual analógica revelou uma curva gaussiana de distribuição normal, com melhora média de 4,44 pontos. A confiabilidade teste-reteste mostrou uma correlação positiva entre a resposta pós-operatória e o mesmo questionário repetido noventa e seis horas depois. A consistência interna foi alta (valor do alfa de Cronbach: pré-operatório = 0,88; pós-operatório = 0,86). Os autores observaram uma melhora significante nas respostas para todas as questões individuais na fase pós-operatória em comparação com a situação pré-operatória (teste *t* de Student – *p* < 0,05).

**Conclusão:**

A versão em português do “*The Utrecht questionnaire for outcome assessment in aesthetic rhinoplasty*” é um instrumento válido para avaliar os resultados do paciente após a cirurgia de rinoplastia.

## Introduction

Rhinoplasty has become one of the main cosmetic surgeries performed by otorhinolaryngologists and plastic surgeons. The major indications for rhinoplasty are: aesthetic and aesthetic-functional.^1^

Most of the studies that discuss aesthetic surgery involve discussions about surgical techniques, access pathways, complications, sequelae and reoperation rates. The evaluation of the final outcome of the was not evaluated from the patient's point of view; this analysis is very important because patient satisfaction is the predominant factor for surgical success.^2^

In rhinoplasty, more than any other aspect of rhinology, patient satisfaction and quality of life should be measures against which the successful procedure must be judged. In this context, quality-of-life questionnaires are very adequate tools that allow the quantitative evaluation of subjective results, such as patient satisfaction and, consequently, the success of the surgery.^3^

Based on such philosophy, Peter Lohuis and colleagues designed a short questionnaire on the basis of a previously validated questionnaire by Alsarraf. “The Utrecht questionnaire for outcome assessment in aesthetic rhinoplasty” contained a visual analogue scale and five simple questions to evaluate subjective body image and quality of life in relation to nasal appearance that influence the satisfaction of the patient undergoing rhinoplasty.^4^, ^5^

The aim of this study was to perform the translation, cross-cultural adaptation and validation of “The Utrecht questionnaire for outcome assessment in aesthetic rhinoplasty” from English to Portuguese of Portugal.

## Methods

Initially, the application for authorization was made to the original author. “The Utrecht questionnaire for outcome assessment in aesthetic rhinoplasty” was translated and adapted according to criteria from Guillemin et al.^6^

The first part of the questionnaire consisted of five questions (E1 through E5) interviewing the patient about body image and quality of life in relation to nasal appearance. Each of the five questions was scored on a five-point Likert scale (1, not at all; 5, very much/often), so that in total a minimum of five points and a maximum of 25 points could be gathered. The third and fourth questions (E3 and E4) were considered “trick” questions that were included with the idea that they provide insight of a disturbance in body perception or body dysmorphic disorder. The second part of the questionnaire consisted of a visual analogue scale on which patients could rate the appearance of their nose on a 10 point scale (0, very ugly; 10, very nice).^4^

**Figure 1:**
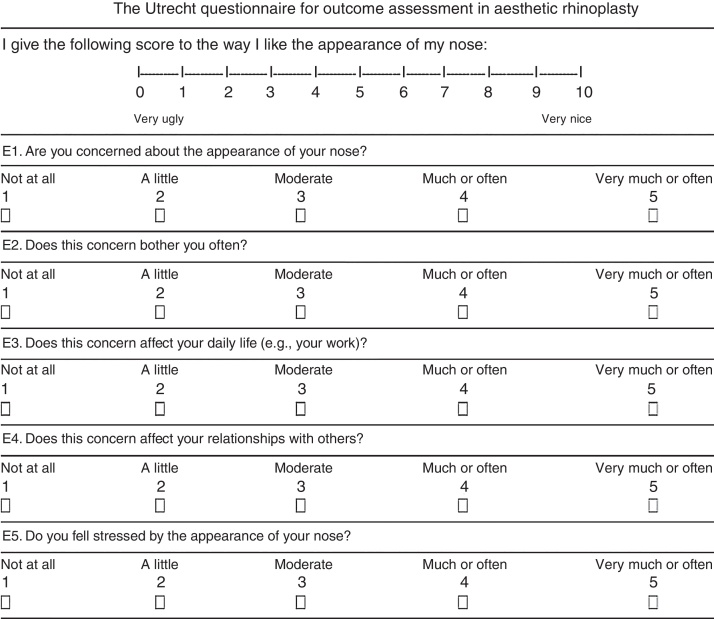
The Utrecht questionnaire for outcome assessment in aesthetic rhinoplasty”.

The original English version (Fig. 1) was delivered to 3 translators with fluency in English but having Portuguese as their native language.

In a second phase, a panel of experts compared the 3 translations and created a consensual translation. This last translation was delivered to 3 translators with English as a native language and with high fluency in Portuguese language, creating retroversion from Portuguese to English. A second panel of experts compared the original version of the questionnaire with the retroversion, and finally an intermediate version was created.

This intermediate version was delivered to 15 patients previously undergoing rhinoplasty. In this way, it was possible to test the comprehension of each item. This phase allowed the cultural adaptation of the version, resulting in the final Portuguese version.

To assess the changes in subjective perception of nasal appearance after surgery, the questionnaire was sent to volunteer patients by e-mail to retrospectively compare the preoperative period with the current situation. Informed consent was obtained from all individual participants included in the study. The inclusion criteria were: patients undergoing primary rhinoseptoplasty in 2015 and 2016, older than 18 years and under 65 years, minimum postoperative period of 6 months and a maximum of 2 years. The exclusion criteria were: patients with congenital facial deformities, who did not speak Portuguese from Portugal and did not intend to participate in the study.

To assess reliability, validity, and internal consistency of our questionnaire, we statistically analyzed preoperative and postoperative questionnaire data. We evaluated test–retest reliability by determining for each question the Pearson correlation coefficient between the postoperative response and the same questionnaire repeated 96 hours later. We used a *t*-test to evaluate these correlation coefficients. The internal consistency of the questionnaire was measured with Cronbach's alpha. The validity of this short questionnaire was assessed by measuring the responsiveness to change. Therefore, we performed a paired *t*-test by comparing preoperative and postoperative responses. For the statistical tests, results with *p* < 0.05 were considered significant.

## Results

**Figure 2:**
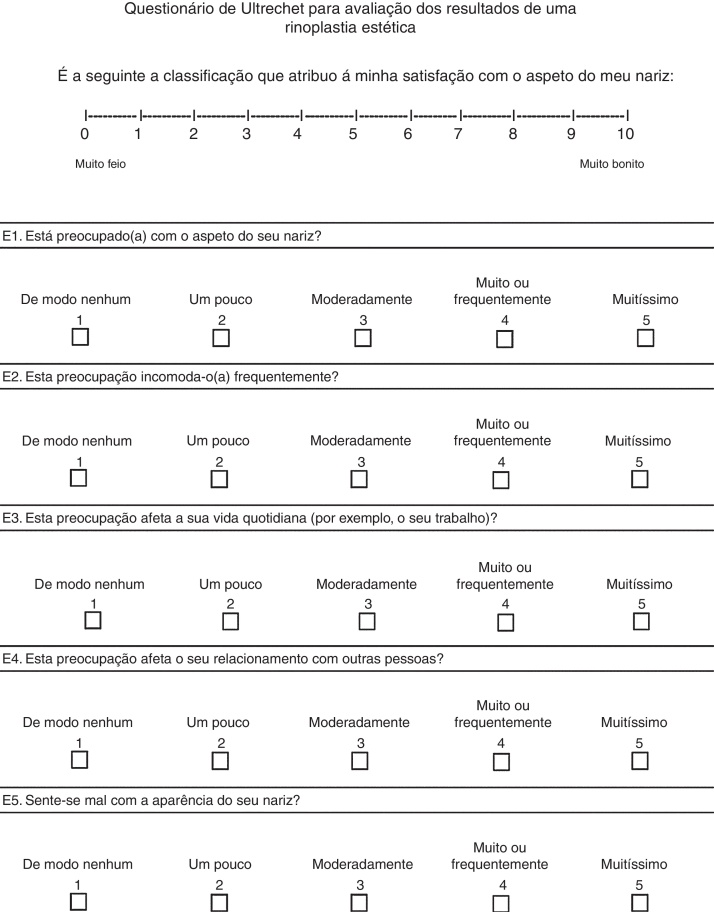
Portuguese final version of “The Utrecht questionnaire for outcome assessment in aesthetic rhinoplasty”.

The final version of the questionnaire translated and adapted from English to Portuguese, according to the Guillemin criteria, is presented in Fig. 2.

We included 50 patients who underwent rhinoplasty for aesthetic or aesthetic-functional reasons. The average age was 37.34 years (Standard Deviation – SD ± 9.96) with a range from 22 to 63 years, 26 (52%) were males and 24 (48%) were females.

**Figure 3:**
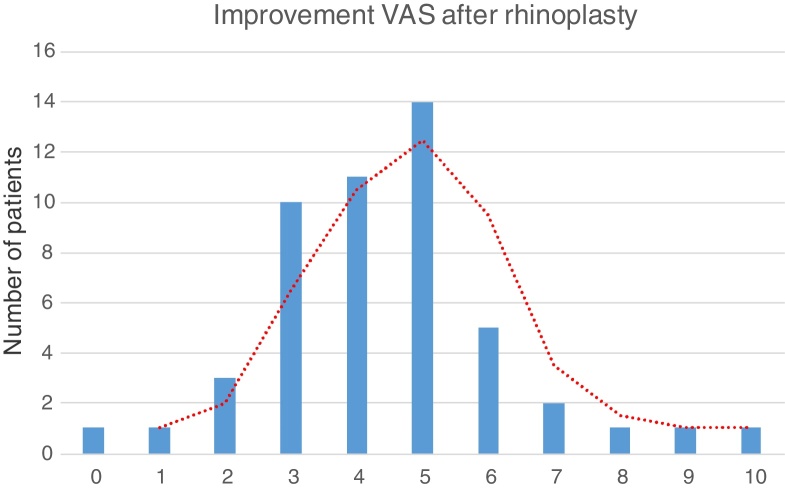
Postoperative improvement on the Visual Analogue Scale (VAS) revealed a Gaussian curve of normal distribution with a mean improvement of 4.44 points.

No patients had negative variation in the score on the Visual Analogue Scale (VAS) comparing preoperative and postoperative appearance (6 months to 2 years after surgery). The postoperative improvement on the visual analogue scale revealed a Gaussian curve of normal distribution with a mean improvement of 4.44 (SD ± 1.8) points. The majority of patients (80%) considered that the nose appearance improved between 3 and 6 points (Fig. 3).

Test–retest reliability measured the stability of an instrument over time after repeated testing. The test–retest reliability showed a positive correlation between the postoperative response and the same questionnaire repeated ninety six hours later (Table 1). The *t*-test evaluation of these correlation coefficients did not show statistically significant differences (*p* < 0.05).

**Table 1:** Test–retest reliability: the Pearson correlation coefficient between the postoperative response and the same questionnaire repeated 96 hours later.

Question (1–5)	Pearson correlation coefficient	*p*
E1	0.89	0.209121
E2	0.87	0.209867
E3	0.91	0.284477
E4	0.86	0.091176
E5	0.87	0.091176
SUM (E1-E5)–(5–25)	0.96	0.098301
VAS	0.89	0.349522

*t*-student test (*p* < 0.05).

Internal consistency referred to the way individual items relate to each other, in order to provide homogeneity among them, and was measured using Cronbach's alpha. The minimum acceptable score for Cronbach's alpha is 0.7. The internal consistency of the questionnaire was adequate. The alpha value was 0.88 for preoperative responses and 0.86 for postoperative responses.

The validity of the questionnaire was assessed by measuring the responsiveness to change. We observed a significant improvement (*p* < 0.05) in response for all individual questions in the postoperative phase as compared with preoperative situation (Table 2).

**Table 2:** Validity of the questionnaire: comparison of preoperative and postoperative scores.

Question (1–5)	Preoperative score	Postoperative score	*p*
E1	3.52	1.68	1.43 × 10^−13^
E2	3.4	1.54	2.23 × 10^−13^
E3	2.08	1.3	4.73 × 10^−13^
E4	2	1.22	7.47 × 10^−13^
E5	3.06	1.38	4.76 × 10^−13^
SUM (E1-E5)–(5–25)	14.06	7.12	1.24 × 10^−12^
VAS	3.82	8.26	1.94 × 10^−12^

*t*-student test (*p* < 0.05).

## Discussion

Some factors may influence patient satisfaction, such as culture, life experience, and especially the patient's expectations about the final outcome, which may or may not be realistic. Although the procedure can often be considered a success by the surgeon, the patient may not feel satisfied with it, and the opposite is also true.^7^, ^8^

Rhinoplasty, altering the patient's image and consequently his/her self-esteem, increasingly suggests the use of satisfaction questionnaires with the procedure.^9^

The easy administration of the questionnaire was one of the concerns that Lohuis and colleagues had in doing it.^4^ We noticed that, after its translation and cross-cultural adaptation, this characteristic was not lost.

The questionnaire was self-administered by e-mail, and just a few minutes are enough to fill out the questions, thus causing the least discomfort for the responder. Probably, this administration of the questionnaire to patients did not alter its purpose, because even if it were administered through interview, the reading would be performed ipsis verbis, without any explanation of the questions. In addition, in our clinical practice patients tend to prefer the questionnaire to be administered by e-mail. This method has some advantages, such as faster completion time, lower rate of missing data and the non-interference of the interviewer's motivation in the responses.^10^

The method used in this study, a retrospective assessment of patient preoperative satisfaction, and prospective evaluation of the patient's postoperative satisfaction, was similar to the one published by other authors.^1^, ^7^

The Portuguese version of the questionnaire showed high internal consistency like the original one, with Cronbach's alpha coefficient higher than 0.8.

The test–retest reproducibility was assessed in different ways. In the original study, patients filled out the questionnaire (self-administration) two times: 1 year after surgery (postoperative response) and 2 to 4 years after surgery (repost operative response). In our study, we evaluated test–retest reliability by computing for each question the Pearson correlation coefficient between the postoperative response and the same questionnaire repeated 96 hours later. Despite differences in the administration of questionnaire, high correlation coefficients have been achieved by both forms.

Regarding the validity of the questionnaire, the Portuguese version revealed an optimum performance and a statistically significant difference in scores was noted when comparing preoperative and postoperative responses. The significant improvement in questions E1 to E5 and in the sum of the questions strongly suggests a postoperative improvement in the subjective perception of nasal appearance and quality of life after rhinoplasty in the study population.

The postoperative improvement on the visual analogue scale revealed a Gaussian curve of normal distribution with a mean improvement of 4.44 points. With this simple tool, such as the visual analogue scale, the analysis of the operated patients can give the surgeon an evaluation of their performance, being this information useful for the surgeon and the patient.

For surgeons who select patient-reported outcome measures to be used in clinical practice, the quality and content of available questionnaires must be considered carefully. These can be divided into three categories: (1) Functional self-assessment; (2) Aesthetic self-assessment (e.g., Utrecht questionnaire); and (3) Aesthetic and functional self-assessment (e.g., Rhinoplasty Outcomes Evaluation). This short and practical questionnaire focuses specifically on aesthetic rhinoplasty. Preoperatively, the questionnaire informs the surgeon about body image and quality of life regarding nasal appearance. Postoperatively, the questionnaire measures the aesthetic result, which, for example, may be useful in deciding whether minor additional corrections are necessary or can be avoided.

The authors concluded that a surgeon performing rhinoplasty can benefit from using this questionnaire in Portuguese. It is simple, quickly completed, and provides important subjective information about the preoperative nasal appearance of the patient and the postoperative surgical outcome.

## Conclusion

The Portuguese version of “The Utrecht questionnaire for outcome assessment in aesthetic rhinoplasty” is a valid instrument to assess results in rhinoplasty patients, resulting good internal consistency, reproducibility and validity.

## Ethical approval

All procedures performed in studies involving human participants were in accordance with the ethical standards of the institution.

This article does not contain any studies with animals performed by any of the authors.

## Previous presentation

This paper was presented as an oral communication in the ENT World Congress IFOS Paris 2017.

## Informed consent

Informed consent was obtained from all individual participants included in the study.

## Conflicts of interest

The authors declare no conflicts of interest.
